# Structural abnormality of the corticospinal tract in major depressive disorder

**DOI:** 10.1186/2045-5380-4-8

**Published:** 2014-09-10

**Authors:** Matthew D Sacchet, Gautam Prasad, Lara C Foland-Ross, Shantanu H Joshi, J Paul Hamilton, Paul M Thompson, Ian H Gotlib

**Affiliations:** 1 Neurosciences Program, Stanford University, Stanford, CA, USA; 2 Department of Psychology, Stanford University, Jordan Hall, Building 01-420, 450 Serra Mall, Stanford, CA 94305, USA; 3 Imaging Genetics Center, Institute for Neuroimaging and Informatics, Keck School of Medicine of USC, Los Angeles, CA, USA; 4 Ahmanson-Lovelace Brain Mapping Center, Department of Neurology, UCLA, Los Angeles, CA, USA; 5 Laureate Institute for Brain Research, Tulsa, OK, USA

**Keywords:** Major depressive disorder (MDD), Diffusion tensor imaging (DTI), Tractography, Clustering, Automated fiber quantification (AFQ), Maximum density path (MDP), Corticospinal tract (CST), Fractional anisotropy (FA)

## Abstract

**Background:**

Scientists are beginning to document abnormalities in white matter connectivity in major depressive disorder (MDD). Recent developments in diffusion-weighted image analyses, including tractography clustering methods, may yield improved characterization of these white matter abnormalities in MDD. In this study, we acquired diffusion-weighted imaging data from MDD participants and matched healthy controls. We analyzed these data using two tractography clustering methods: automated fiber quantification (AFQ) and the maximum density path (MDP) procedure. We used AFQ to compare fractional anisotropy (FA; an index of water diffusion) in these two groups across major white matter tracts. Subsequently, we used the MDP procedure to compare FA differences in fiber paths related to the abnormalities in major fiber tracts that were identified using AFQ.

**Results:**

FA was higher in the bilateral corticospinal tracts (CSTs) in MDD (*p*’s < 0.002). Secondary analyses using the MDP procedure detected primarily increases in FA in the CST-related fiber paths of the bilateral posterior limbs of the internal capsule, right superior *corona radiata*, and the left external capsule.

**Conclusions:**

This is the first study to implicate the CST and several related fiber pathways in MDD. These findings suggest important new hypotheses regarding the role of CST abnormalities in MDD, including in relation to explicating CST-related abnormalities to depressive symptoms and RDoC domains and constructs.

## Background

Major depressive disorder (MDD) is the most common psychiatric disorder in the United States [1], accounting for approximately half of disability-adjusted life years worldwide [2], with major economic and personal costs [3]. MDD involves a wide range of symptoms, including most prominently negative affect and anhedonia (loss of pleasure), as well as difficulties in psychomotor functioning, sleep, and weight changes. If we could better understand the neural basis of MDD, we may be able to better prevent and treat this debilitating disorder.

Across different areas of neuroscience, there has been growing interest in delineating brain networks, in contrast to examining specific brain regions in isolation. Networks of brain regions have been increasingly implicated in depressive pathology, underscoring the need to understand depression-related anomalies in the connections among these regions [4-6]. In this context, diffusion-weighted imaging can assess diffusion properties of white matter and can be used to infer brain connectivity. Using diffusion tensor imaging (DTI), water diffusion can be quantified using fractional anisotropy (FA), which measures the degree of directional preference in water diffusion. FA, the most commonly used diffusion metric, is influenced by intra-voxel orientation dispersion, axonal myelination and packing density, membrane permeability, the number of axons, and partial volume effects [7]. Moreover, tractography algorithms can use diffusion tensor information to estimate the location and direction of fiber tracts. DTI has been used to characterize abnormal white matter diffusion properties in a range of diseases, including psychiatric disorders involving psychosis and disturbances in mood and attention [4,5,8,9].

To our knowledge, there have been three reviews documenting diffusion abnormalities in MDD [4-6]. Across these three reviews, there has been considerable discrepancy in the direction and location of effects of white matter abnormalities in MDD. This may be because of significant heterogeneity in participant samples (e.g., half of the studies included in one review assessed elderly individuals [5]), meta-analytic methods (e.g., qualitative [5], signed differential mapping (SDM) [6], and activation likelihood estimation (ALE) [4]), individual study analysis techniques (e.g., tractography, voxel-based analysis (VBA), or tract-based spatial statistics (TBSS)), and/or study inclusion criteria (e.g., only analyzing decreases in FA [6]). Thus, our current understanding of white matter pathology in MDD is based on relatively few studies that, themselves, incorporate heterogeneous methodological approaches. Most studies of diffusion in MDD have not assessed tractography, but instead examined FA or other diffusion measures in specific regions of interest (ROIs), or, globally, using VBA or TBSS [4-6]. Tractography uses directional information from the diffusion data to extract diffusion properties from specific fiber tracts, and it may offer greater power to detect disease-related abnormalities than do VBA and TBSS [10].

Few studies have used tractography-based methods to characterize white matter connectivity in MDD. Zhang et al. first used tractography to identify the cingulum bundle and uncinate fasciculi and then estimated diffusion properties in these fiber tracts. These investigators found that FA was lower and mean diffusivity was higher in the right uncinate fasciculus in depressed individuals relative to nondepressed controls [10]. In a second study, Zhang and colleagues found MDD-related reductions in FA in tractography-identified anterior limb of the internal capsule, an important component of the cortico-striatal-pallidal-thalamic (CSPT) circuit [11]. Finally, in a connectomics framework, tractography and graph theory have been used to explicate large-scale network abnormalities in depression [12,13].

Whole-brain tractography commonly includes tens of thousands of fibers; consequently, findings using this technique in isolation can be difficult to interpret. To better understand such massive amounts of data, whole-brain tractography is often summarized. One data reduction method identifies key fiber tracts by requiring manual tracing of an ROI which is followed by algorithmic assessment of the fibers that pass through it (as in [10,11]). This manual identification of ROIs is time consuming, however, and limits the number of fascicles that can be assessed. In addition, manual tracing methods can introduce investigator bias during selection and tracing of ROIs. In contrast, clustering methods permit the automated, unbiased summarization of fiber tract information, by using anatomical and DTI information to locate important fiber tracts. Automated fiber quantification (AFQ) [14] and the maximum density path (MDP) [15] approach are two such clustering methods. Briefly, AFQ identifies important white matter tracts by assessing sets of fibers that intersect pairs of waypoint ROIs. Similarly, the MDP procedure uses a graph search method in a set of white matter ROIs to identify abnormalities in fiber paths. MDPs are smaller and more numerous than the AFQ-identified tracts and provide complementary anatomical information.

Given the likely importance of anomalies in white matter connectivity in MDD, the inconsistency in the literature concerning diffusion-related findings in this disorder, and the recent development of sensitive, automated, tractography clustering methods, the present study was designed to use the AFQ and MDP tractography clustering methods to automatically characterize properties of white matter diffusion in MDD. First, we used AFQ to identify depression-related anomalies in FA in 18 major white matter paths. MDPs allow for additional and complementary information relative to tract properties derived by AFQ, given their small size, large number, and association with major white matter tracts. After identifying abnormal white matter pathways using AFQ, we conducted secondary analyses in a subset of MDPs that were associated with these specific paths. In addition, given evidence that age of onset of depression and severity of disorder are related to abnormalities in white matter properties [6,16], we assessed the relations between these two variables as well as the level of global functioning and diffusion properties of abnormal white matter paths.

Thus, we used information from tractography and took advantage of the lower bias and higher efficiency of two automated clustering methods to study major white matter paths in MDD. We hypothesized that FA would be lower in depressed individuals in the uncinate fasciculus, which links regions associated with emotion processing (e.g., hippocampus, amygdala) with regions implicated in cognitive control (e.g., prefrontal cortex).

## Methods

### Participants

Participants were 14 women diagnosed with MDD and 18 healthy, age-matched, female controls (CTLs) 18–55 years of age. The Structured Clinical Interview for DSM-IV-TR Axis I (SCID-I) [17] was used to establish a psychiatric diagnosis of MDD based on DSM-IV-TR criteria. To qualify for study entry, individuals in the CTL group could not have met criteria for any past or current DSM-IV-TR Axis I disorder. Exclusion criteria for both MDD and CTL participants included current alcohol or substance abuse or dependence and head trauma resulting in loss of consciousness greater than 5 min. During the SCID-I, to assess age of onset of depression, depressed participants were asked at what age they first experienced a depressive episode. A trained interviewer also completed the Global Assessment of Functioning (GAF) scale [18]. This scale indexes, from 1 to 100 (sickest to healthiest), the level of the participants’ social, occupational, and psychological functioning. Severity of depression was assessed with the Beck Depression Inventory-II (BDI-II [19]). The Stanford University Institutional Review Board approved the study and informed consent was collected from each participant.

### MRI data acquisition

Whole-brain diffusion-weighted and high-resolution T1-weighted images were collected using a Discovery MR750 3.0 T MR system (GE Medical Systems, Milwaukee, WI, USA), housed at the Stanford Center for Neurobiological Imaging. The T1-weighted images were used for anatomical registration (spoiled gradient echo (SPGR) pulse sequence; repetition time (TR) = 6,240 ms; echo time (TE) = 2.34 ms; flip angle = 12°; resolution = 0.9 mm isotropic; 186 slices; scan duration = 5 min 15 s). The diffusion-weighted scan was a single-shot, dual-spin-echo, echo-planar imaging sequence (96 unique directions; *b* = 2,000 s/mm^2^; TR = 8,500 ms; TE = 93.6 ms; resolution = 2 mm isotropic; 64 slices; scan duration = 15 min 1 s). Nine non-diffusion-weighted (*b* = 0 s/mm^2^) volumes were additionally collected for anatomical localization and registration purposes.

### AFQ procedure

AFQ systematically uses whole-brain tractography methods to characterize major white matter fiber tracts. Here we briefly describe the AFQ procedure (see Additional file 1 for more detail). First, diffusion data were preprocessed, including motion correction, data alignment, resampling, and trilinear interpolation [20]. Tensors were then fit at each voxel using a robust tensor fitting method [21], and FA was computed as the normalized standard deviation of the tensor’s eigenvalues. FA ranges from 0 (perfectly isotropic) to 1 (perfectly anisotropic diffusion). Following this, tractography was estimated using a deterministic streamline tracing algorithm [22,23]. Then, waypoint ROIs labeled on the MNI template were warped into participant-specific diffusion space, and fibers intersecting these ROIs were identified. After a series of fiber cleaning and tract refinement steps, the central portion of each fiber tract was located and diffusion metrics were computed along this core, resulting in a “tract profile”. These tract profiles permit the systematic and unbiased assessment of group differences in diffusion metrics, FA in this study. After identifying tract profiles, we computed the mean FA along each white matter tract.

### MDP procedure

The MDP approach allows for the automated assessment of compact and localized white matter paths on an individual-participant basis [15] (see Additional file 1 for more detail). Because MDPs are smaller, more numerous, and associated with major white matter tracts (i.e., they are located in major tracts or in areas to which these tracts project), they offer additional information to that obtained using AFQ. We identified MDPs in 50 white matter regions described in the Johns Hopkins University white matter atlas, resulting in a total of 67 MDPs (several regions have more than one MDP). To implement this procedure, we first corrected the diffusion data for eddy current and motion effects; next, we estimated whole-brain tractography using an optimized global probabilistic tractography method [24]. Then, from the whole-brain tractography computed using the global method, we created fiber density images for each white matter ROI by identifying fibers that intersect with the ROI (AFQ-identified fibers intersected pairs of ROIs). The next step employed graph theoretical analysis. Specifically, fiber density graphs were created with nodes as voxel locations and edges as density information. Seed points identified in the white matter atlas were then warped into each fiber density graph image. Using an optimized grid search method to find the path of highest density [25], MDPs were identified between each pair of seed points. The resulting paths were compact representations of the given tract’s scale/size, location, and geometry/shape. Finally, paths were registered spatially across individuals using a geodesic curve registration procedure [26,27], allowing us to conduct between-group comparisons of FA in a point-wise manner.

### Analysis plan and statistical analysis

In the first stage of analysis, we used two-sample *t*-tests to compare the 18 AFQ-identified fiber tract core mean FA values for the MDD and CTL groups. To correct for false positive inflation as a result of multiple comparisons, we implemented a false discovery rate (FDR) procedure (*q* = 0.05) [28]. This analysis identified major fiber bundles in which there were depression-related abnormalities. Using Pearson linear partial correlation (controlling for age), we assessed the correlations between identified abnormal fiber tracts and age of onset of depression, severity of depression (BDI-II scores), and level of global functioning (GAF scores) in the MDD group.

Second, we identified MDPs that were associated (i.e., overlapping, outside of the AFQ fiber tract but in projection fibers, or spatially proximal) with the abnormal fiber tracts that were identified using AFQ. Spatially proximal MDPs were included because AFQ incorporates weighted FA values into the estimates of major fiber tract FA from fibers that are not in the fiber tract core; thus, proximal MDPs may exhibit relevant FA abnormalities. The step-wise analysis procedure was implemented because MDPs are neuroanatomically associated with the AFQ fiber tracts, and are smaller and more numerous, therefore allowing for additional but complementary information to that provided by the AFQ-identified major fiber tracts. We conducted two-sample *t-*tests to assess point-wise differences between the MDD and CTL groups, using FDR to correct for multiple comparisons across points for each analyzed MDP (i.e., the subset of the 67 MDPs that were included for further analysis given their relation to the abnormal fiber tracts identified using AFQ).

## Results

### Demographic and clinical characteristics

Means and standard deviations for demographic and clinical variables for the 14 depressed and 18 control female participants are presented in Table 1. The two groups did not differ in age (*t*(30) = -1.53, *p* > 0.10), handedness (*χ*^2^(1) = 0.14, *p* > 0.10), or level of education achieved (*t*(30) = -1.34, *p* > 0.10). As expected, the depressed participants had significantly higher BDI-II scores than did the never-depressed controls. Half of the participants in the depressed group met criteria for at least one anxiety disorder, and three depressed participants were currently taking psychotropic medications (see Table 2).

**Table 1:** Participant demographic and clinical characteristics

	**CTL (*N*** **= 18)**			**MDD (*N*** **= 14)**			***p *** **value**
Age in years (*M* | *SD* | min/max)	30.4	10.2	18.9/52.1	35.6	8.4	22.8/48.5	>0.10^b^
Education level^a^ (*M* | *SD* | min/max)	6.6	1.5	4/8	7.2	1.1	4/8	>0.10^c^
BDI-II (*M* | *SD* | min/max)	2.2	3.2	0/11	31.7	6.6	22/43	<0.001^b^
Global Assessment of Functioning (*M* | *SD* | min/max)	87.8	7.0	75/99	53.0	6.6	35/60	<0.001^b^
Age of onset (*M* | *SD* | min/max)	NA			16.3	6.8	3/26	NA
Handedness (right | left)	16		2	13		1	>0.10^c^

*BDI-II* Beck Depression Inventory-II, *GAF* Global Assessment of Functioning, *M* mean, *SD* standard deviation, *MDD* major depressive disorder, *CTL* control.

^a^Level of education was assigned as follows: having finished 1) elementary school, 2) junior high school, 3) high school, 4) some college, 5) technical school, 6) junior college, 7) 4-year college, or 8) graduate or professional education.

^b^Computed using two-sample *t*-test.

^c^Computed using chi-square test.

**Table 2:** Current comorbid diagnoses and psychotropic medications of the MDD participants

	**Participants**	**% of total**
Comorbidities	7	50
Social phobia	4	28.6
General anxiety disorder	3	21.4
Panic disorder	2	14.3
Post-traumatic stress disorder	2	14.3
Specific phobia	2	14.3
Bulimia nervosa	1	7.1
Psychiatric medications	3	21.4
Aripiprazole	2	14.3
Duloxetine	1	7.1
Gabapentin	1	7.1
Quetiapine	1	7.1
Sertraline	1	7.1
Venlafaxine	1	7.1

*MDD* major depressive disorder.

### AFQ

**Figure 1:**
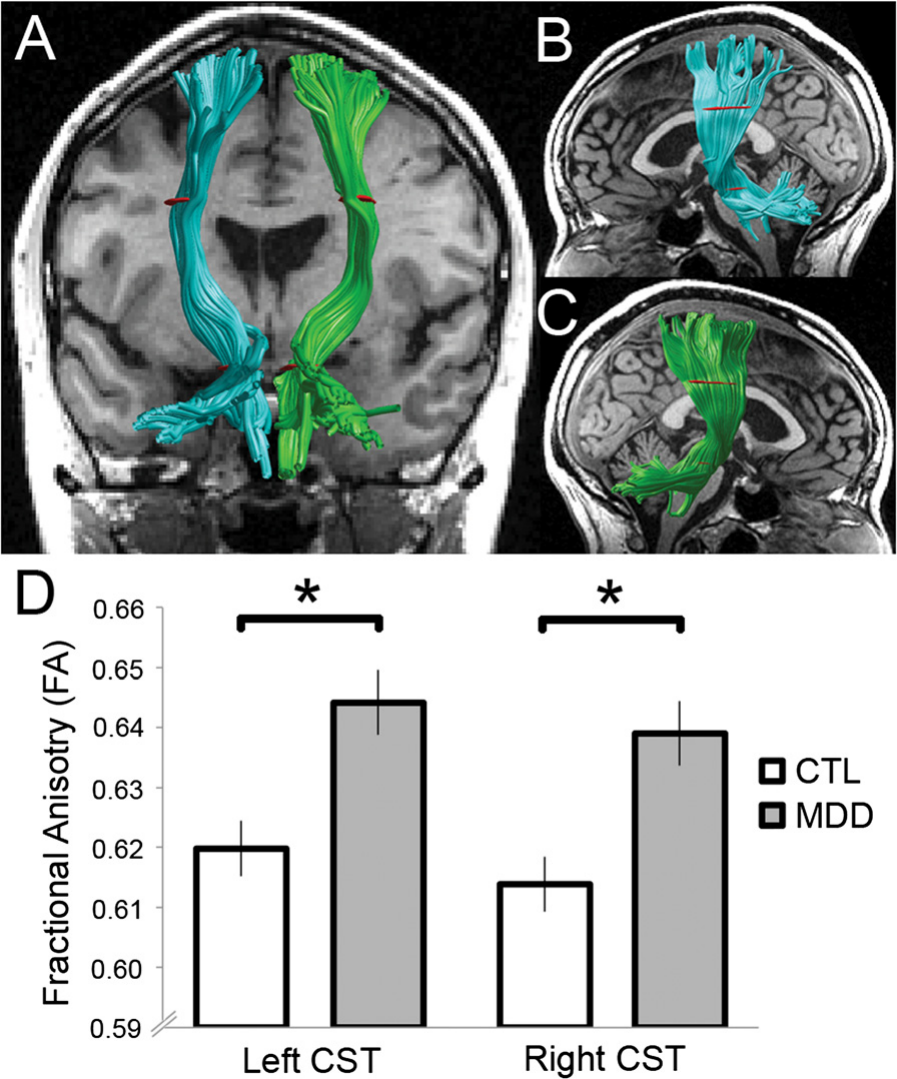
**AFQ corticospinal tracts.** Corticospinal tracts (CSTs) rendered for an example subject. Three hundred fibers were rendered for each tract. The left CST is colored *teal* and the right CST *green*. Waypoint regions of interest (ROIs) are depicted in *red*. Fractional anisotropy (FA) computed for the fiber tract core, between the waypoint ROIs, was meaned and compared between groups. **(A)** Bilateral CSTs viewed posteriorly with a T1-weighted coronal slice at the anterior commissure. **(B)** Left CST and **(C)** right CST viewed laterally with a mid-sagittal T1-weighted slice. **(D)** Group differences in mean CSTs. *Asterisks* indicate statistical significance from two-sample *t-*tests between groups. *Error bars* represent standard error of the mean (SEM). See Table 4 for group means and standard deviations. *AFQ* automated fiber quantification, *CTL* control group, *MDD* depressed group.

Across the entire sample, AFQ was unable to characterize 5 of the 576 fiber tracts (i.e., *N* × [number of fiber tracks] = 32 × 18): the callosum forceps major for three participants and the callosum forceps minor for two participants. These participants were excluded from analyses involving these particular fiber tracts. The failure of AFQ to identify these fibers may be a result of crossing fibers, noise in the data, abnormal anatomy that caused problems for automated segmentation, or small fiber tracks for which it is difficult to compute statistics. Of the 18 analyzed fiber groups (Table 3), two distinguished MDD from CTL participants after correcting for multiple comparisons: the left corticospinal tract (CST) (*t*(30) = 3.45, *p* < 0.002) and the right CST (*t*(30) = 3.79, *p* < 0.001) (Table 4, Figure 1). Both CSTs were characterized by greater FA in the MDD than in the CTL group (Table 4). As an exploratory analysis, we divided the group of MDD participants into two subgroups based on the presence or absence of comorbid anxiety; these two subgroups did not differ in FA for either the left or the right CST (*p*’s > 0.10). In addition, the group differences in CST were unchanged after removing the three MDD participants who were taking psychotropic medications (left CST: *t*(27) = 3.45, *p* < 0.002; right CST: *t*(27) = 3.11, *p* < 0.005). Finally, in the MDD group, we correlated age of onset and severity of depression, and level of global functioning, with mean FA individually for both left and right CST. No significant correlations were obtained (*p* > 0.10).

**Table 3:** AFQ-identified fiber tracts

**Laterality**	**AFQ fiber tracts**
L/R	Thalamic radiation
L/R	Corticospinal
L/R	Cingulum cingulate
NA	Callosum forceps major
NA	Callosum forceps minor
L/R	IFOF
L/R	ILF
L/R	SLF
L/R	Uncinate
L/R	Arcuate

*AFQ* automated fiber quantification, *L* left, *R* right, *IFOF* inferior fronto-occipital fasciculus, *ILF* inferior longitudinal fasciculus, *SLF* superior longitudinal fasciculus.

**Table 4:** Group differences in FA in AFQ-identified fiber tracts

**AFQ fiber group**	**CTL FA**		**MDD FA**		***p *** **value**
	***M***	***SD***	***M***	***SD***	
Left corticospinal tract	0.620	0.020	0.644	0.020	<0.002
Right corticospinal tract	0.614	0.021	0.639	0.015	<0.001

*p* values were computed using two-sample *t*-tests and a false discovery rate (FDR) procedure was used to account for false positives as a result of multiple comparisons. See Figure 1 for corticospinal tract (CST) renderings and graphical display of differences.

*AFQ* automated fiber quantification, *MDD* major depressive disorder, *CTL* control, *FA* fractional anisotropy, *M* mean, *SD* standard deviation.

### MDPs

For the second stage of the analysis plan, we identified MDPs associated with the AFQ-identified CST. This resulted in the identification of 24 unique MDPs from seven white matter ROIs (from a total of 50). The 24 MDPs (12 in each hemisphere) comprised 35.8% of the total set of 67 MDPs (Table 5). These MDPs overlap, are along the same white matter bundle, or are spatially proximal to the CST projections identified in the AFQ analysis.

**Table 5:** MDP locations

**White matter region**	**MDPs per hemisphere**
Anterior limb of the internal capsule	2
Posterior limb of the internal capsule	2
External capsule	1
Corticospinal tract	1
Anterior *corona radiata*	2
Superior *corona radiata*	2
Posterior *corona radiata*	2

White matter regions of interest from the Johns Hopkins University Atlas and the number of MDPs per region, per hemisphere. A total of 24 MDPs were assessed.

*MDP* maximum density path.

**Figure 2:**
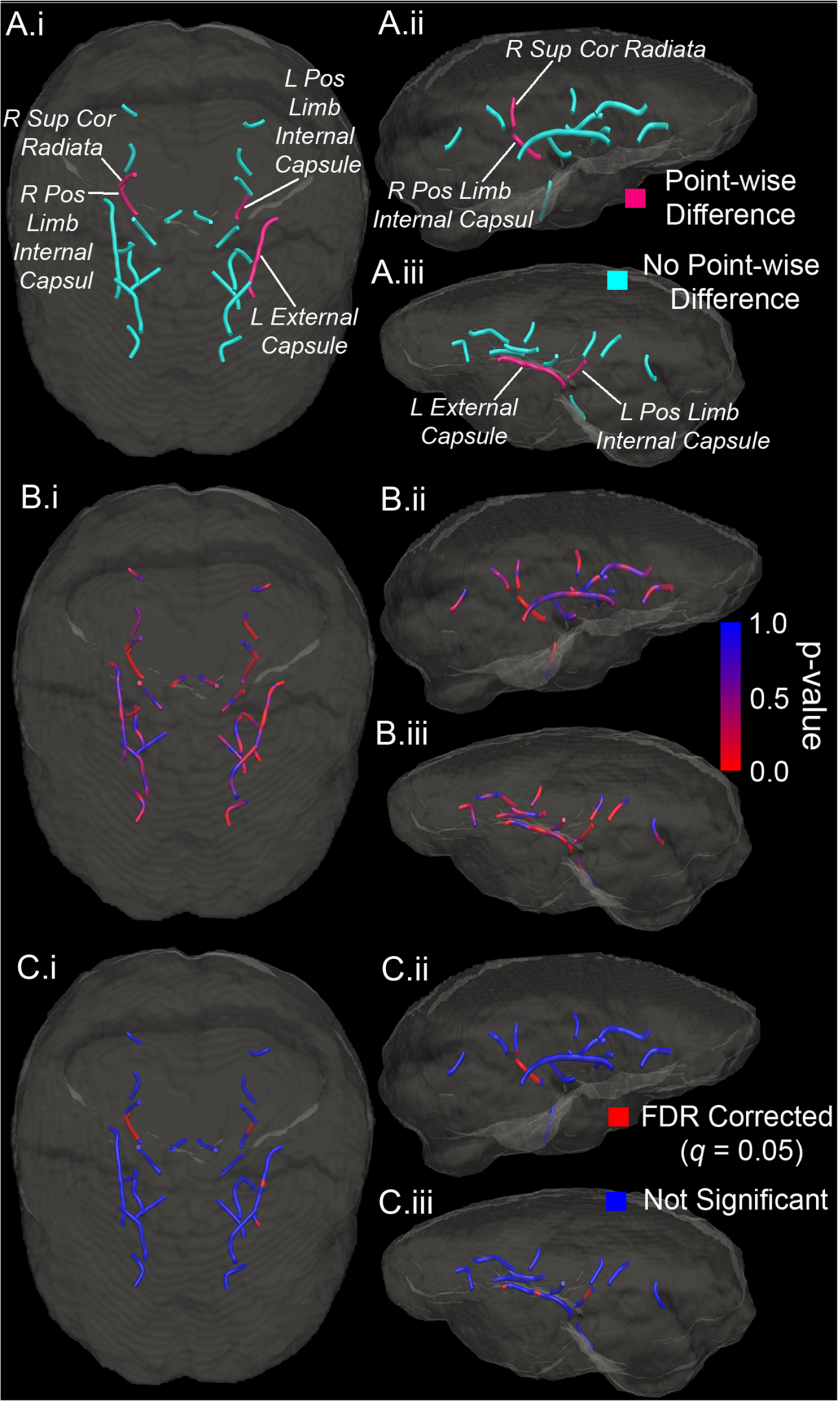
**Group differences in MDPs. (A)** Rendering of analyzed MDPs associated with the corticospinal tract (CST). A.i Superior view. A.ii Right lateral view. A.iii Left lateral view. MDPs that exhibited point-wise differences (right superior *corona radiata*, right posterior limb of the internal capsule, left posterior limb of the internal capsule, left external capsule) are labeled and colored *magenta*. **(B)** Rendering of analyzed MDPs with point-wise *p* values computed from two-sample *t*-tests. *Red* indicates lower *p* values and greater significance. **(C)** Rendering of analyzed MDPs with FDR-corrected point-wise statistical tests. *Red* indicates significant test. *MDP* maximum density path, *FDR* false discovery rate, *R* right, *L* left, *Sup* superior, *Pos* posterior, *Cor corona*.

We conducted two-sample *t*-tests on a point-by-point basis along each of the identified MDPs. After correcting for multiple comparisons by using FDR independently for each MDP, the MDD and CTL groups exhibited point-wise differences in four MDPs: the left posterior limb of the internal capsule, the right posterior limb of the internal capsule, the right superior *corona radiata*, and the left external capsule (Table 6, Figure 2). Because each of the four MDPs was located in a unique region, and because each of these regions included two analyzed MDPs, only one of the two MDPs identified for each implicated white matter region yielded point-wise group differences after FDR correction. Of the identified points that differed between groups (27 total across the four MDPs), all except three of the six points of the left external capsule were characterized by greater FA in the MDD than in the CTL group.

**Table 6:** Group FA differences in MDPs

**White matter region**	**Total points in MDP**	**Group differences**	**% of points**
Left posterior limb of the internal capsule	16	6	37.5
Right posterior limb of the internal capsule	17	14	82.4
Right superior *corona radiata*	17	1	5.9
Left external capsule	48	6	12.5

Group differences were assessed using two-sample *t*-tests, and a false discovery rate (FDR) procedure was used to account for false positives as a result of multiple comparisons.

*FA* fractional anisotropy, *MDP* maximum density path.

## Discussion

The literature examining white matter abnormalities in MDD is methodologically varied and sparse and has yielded inconsistent findings. In this context, the current study was designed to capitalize on the improved detection power of tractography, the reduced bias of automated clustering methods, and more systematic and data-driven analysis, to assess abnormalities in white matter in MDD and begin to yield a more systematic and reliable connectomics of depression. Indeed, this is the first study to use automated tractography clustering to characterize white matter in depression. Our analyses included fiber tracts that have been previously studied in this disorder, in addition to several tracts that have not before been examined.

Using AFQ [14], we found that MDD was characterized by abnormalities in FA in the bilateral corticospinal tracts. We then used the MDP procedure [15] to further probe localized abnormalities that were associated with these group differences. This analysis revealed, for the first time, increased FA in the bilateral posterior limbs of the internal capsule, right superior *corona radiata*, and the left external capsule in MDD.

Previous studies have primarily documented reduced FA associated with MDD. In contrast, the current results include almost exclusively *increased* FA in participants diagnosed with this disorder. This discrepancy may be a result of the small number of studies considered in the previous reviews (12 studies [5], 11 studies [4], 7 studies [6]). In addition, Liao et al. only analyzed data indicating increases in FA in MDD [4]; the two quantitative reviews [4,6] excluded tractography studies; and the third review [5] included only one tractography study in the discussion of MDD. Because tractography incorporates directionality information that is used to identify important white matter pathways, it may allow for greater detection power than do whole-brain voxel-wise techniques (e.g., VBA or TBSS) and, thus, may explain why previous research did not report increases in FA in CST in MDD. It is also possible that the automated clustering methods that we implemented yield greater spatial specificity than do earlier methods, and that decreases in FA in MDD that were previously identified using VBA and TBSS were, in fact, inaccurately reported to be localized to major fiber tracts.

It is important to also note that other investigators have reported depression-related increases in FA. For example, Blood et al. found that the ventral tegmental area was associated with greater FA in MDD than in control participants [29]. Moreover, several studies have identified regions of increased FA in bipolar disorder (BD) [5] in areas of the corpus callosum [30] and the frontal lobe [31], including the uncinate fasciculus, optic radiation, and anterior thalamic radiation [32]. Given recent interest in examining transdiagnostic factors in the proposed NIMH RDoC framework, it will be important in future research to investigate how tract-specific FA may correspond to signs and symptoms of disorders of emotion and mood with respect to specific RDoC domains and constructs.

Previous research has suggested that increased FA of the CSTs is related to decreases in FA of the superior longitudinal fasciculi (SLF). Specifically, Douaud et al. reported increases in CST FA in individuals with mild cognitive impairment and Alzheimer’s disease compared to healthy controls; moreover, using a method of quantitative crossing fiber tractography, Douaud et al. found that the increases in CST FA were associated with reduced FA of SLF association fibers in a crossing fiber region at the level of the centrum semiovale [33]. Although these findings raise the intriguing possibility that MDD-related increases in FA of the CST are related to selective sparing of this tract with concurrent abnormality in the SLF, we did not find SLF abnormalities in the current study. Future research using imaging and tractography methods that allow for greater resolution of crossing fibers may permit a better assessment of whether increases in CST FA in MDD are related to abnormalities in regions of crossing fibers.

Given the role of the CSTs in motor processes, it is possible that our findings of anomalous FA in these structures are related to psychomotor symptoms that often characterize MDD [34]. More specifically, motor retardation and agitation, criterion symptoms of MDD, may result from aberrations in white matter microstructure connecting the brainstem to motoric regions of cortical gray matter [35]. The current findings offer a foundation from which future research might explore this hypothesis. Importantly, although the white matter of primate CSTs is understood to arise primarily from the primary motor cortex, projections from the somatosensory, cingulate, and insular cortices are also represented [35]. Thus, the CST is likely to be involved in a variety of functions and thus may be related to a range of depression-related functioning (e.g., somatosensory, affective, and cognitive). Future research, therefore, may profitably assess the relations of these important symptom domains with CST diffusion properties in MDD. Moreover, given the varied projection profile of the CST, future research should assess relations between abnormalities in CST FA and gray matter properties (e.g., volume) in this disorder.

Although controversial, findings of abnormal fronto-striatal networks in MDD have led to the formulation that this disorder is a “disconnection syndrome” characterized by reduced connectivity between cortical and subcortical brain regions [4,5,36]. Evidence for this formulation includes observations that frontal white matter FA is reduced in MDD [37] and is correlated with remission from depression [38]. The current findings provide evidence that MDD may be characterized by abnormalities in connectivity between subcortical and brainstem structures and cortical gray matter regions. In future studies, investigators might use the AFQ and MDP procedures to examine the viability of the disconnection syndrome formulation more systematically, given that these procedures yield increased spatial specificity in neuroanatomical abnormalities associated with MDD.

The uncinate fasciculus and thalamic radiation are the most commonly studied fiber tracts in mood disorders [5]; indeed, we had hypothesized that we would find abnormalities of the uncinate fasciculus in MDD. The interest in these white matter tracts is due primarily to their potential involvement in abnormal cognitive control over emotion processing. Specifically, the uncinate fasciculus includes connections between medial temporal lobe regions associated with emotion processing (e.g., the hippocampi and amygdala) and the frontal cortex (involved in cognitive control); similarly, the white matter of the thalamic radiation links the frontal cortex with the thalamus (potentially a key connection in the disconnection syndrome formulation). Notably, we did not find abnormalities in these two tracts. This may be due in part to the location of the default AFQ waypoint ROIs, which are placed to identify the fiber tract cores and, thus, limit the assessment of variability more proximal to cortex.

The current results indicate that AFQ and MDPs are complementary techniques for the quantification and characterization of white matter paths in psychiatric populations and represent an important step towards the automated and efficient characterization of psychopathology, as demonstrated here in MDD. Given the sensitivity and automated nature of these methods, they may prove useful in identifying and characterizing biomarkers that can facilitate efforts to prevent and treat psychiatric disorders.

Despite the strengths of these procedures, we should note three limitations of the present study. First, the sample size in the current study was relatively small; thus, it is possible that the analyses are underpowered to find reductions in FA that have been previously reported, or significant relations between CST FA and age of onset or severity of depression, or level of global functioning. Second, our sample of depressed participants was heterogeneous with respect to the presence of anxiety comorbidities and medication use. We did not find differences in CST FA between the comorbid and non-comorbid participants in the MDD sample, nor did the effects we report appear to be determined by psychotropic medication use in a small subset of our depressed sample. Thus, it seems that these factors did not confound our results. Third, as with all FA-related results, the biological basis of the observed abnormality is unclear, as many factors can influence this metric: the level of orientation dispersion, myelination, numbers of axons, membrane permeability, axonal packing density, geometric properties of the tract, partial volume effects, and influences from branching, merging, or crossing fibers [7].

## Conclusions

Using tractography clustering methods, we identified abnormalities in major white matter paths in MDD, specifically in the CSTs and several related pathways, including bilateral posterior limb of the internal capsules, right superior *corona radiata*, and left external capsule. These are the first results to implicate abnormality of the CST and related pathways in MDD. These findings highlight important future research directions, including increasing our understanding of CST abnormalities in the context of depressive symptoms and in relation to RDoC domains and constructs. Finally, the current study demonstrates that tractography clustering techniques can be used to increase our understanding of white matter abnormalities in MDD.

## Abbreviations

AFQ: Automated fiber quantification; ALE: Activation likelihood estimation; BD: Bipolar disorder; CSPT: Cortico-striatal-pallidal-thalamic; CST: Corticospinal tract; CTL: Control; DSM-IV-TR: Diagnostic and Statistical Manual of Mental Disorders 4th Edition, Text Revision; DTI: Diffusion tensor imaging; FA: Fractional anisotropy; FDR: False discovery rate; MDD: Major depressive disorder; MDP: Maximum density path; MNI: Montreal Neurological Institute; MR: Magnetic resonance; NIMH: National Institute of Mental Health; RDoC: Research domain criteria; ROI: Region of interest; SCID: Structured Clinical Interview for DSM-IV-TR Axis I; SDM: Signed differential mapping; SLF: Superior longitudinal fasciculus; SPGR: Spoiled gradient; TBSS: Tract-based spatial statistics; TE: Echo time; TR: Repetition time; VBA: Voxel-based analysis.

## Competing interests

The authors declare that they have no competing interests.

## Authors’ contributions

MDS conceived of the project, analyzed the data, interpreted the findings, and drafted the manuscript. GP analyzed the data, interpreted the findings, and drafted the manuscript. LCFR coordinated the study, interpreted the findings, and drafted the manuscript. SHJ contributed to the MDP analysis and statistical methods. JPH acquired the data, interpreted the findings, and drafted the manuscript. PMT interpreted the findings and drafted the manuscript. IHG coordinated the study, interpreted the results, and drafted the manuscript. All authors read and approved the final manuscript.

## Supplementary Material

Additional file 1 **Supplementary information for AFQ and MDP methods.** File contains additional information regarding implementations of the AFQ and MDP methods. Click here for file
