# Supplementary material for: Structural abnormality of the corticospinal tract in major depressive disorder
Source: Biol Mood Anxiety Disord. 2014 Sep 10;4:8. doi: 10.1186/2045-5380-4-8 (PMC4187017; doi:10.1186/2045-5380-4-8)
Supplement: Additional file 1 — Supplementary information for AFQ and MDP methods. File contains additional information regarding implementations of the AFQ and MDP methods. [file 2045-5380-4-8-S1.docx]

**Additional file 1**

*AFQ: MRI Preprocessing*

Analyses were conducted primarily using Matlab (The MathWorks, Natick, MA, USA). Openly available software (MrDiffusion and AFQ; http://vistalab.stanford.edu/) was used for all AFQ-related analyses, including the steps of preprocessing, tractography, and fiber tract identification. Subject motion effects were reduced using 6-parameter rigid-body realignment. Diffusion-weighted images were registered to a mean image created by averaging all non-diffusion weighted (i.e., *b* = 0) images. The mean non-diffusion weighted image was aligned to the high-resolution T1-weighted image. Resampling of the diffusion-weighted data then commenced by combing the transforms from the motion correction and anatomical registration into an omnibus transform, followed by trilinear interpolation [1]. Orientations of the diffusion directions were maintained by applying the rotation components of the omnibus transform to the gradient directions.

We then implemented a robust tensor fitting method to estimate tensors [2]. RESTORE reduces noise from spatially and temporally varying artifacts (e.g., motion, cardiac pulsation) by iteratively removing outlier data identified using reweighted least-squares regression. Eigenvalues index the magnitude of the major axes of an ellipsoid. FA was computed on a voxel-wise basis by taking the normalized standard deviation of the three eigenvalues identified by eigenvalue decomposition of the estimated diffusion tensor [3].

*AFQ: Computing Fiber Tract Profiles*

Here we describe the AFQ processing pipeline (see [4] for additional details). We used AFQ v0.1 to identify 20 major fiber tracts on an individual subject basis. Four fiber tracts were discarded based on known inconsistencies in automatic identification caused by failure of deterministic tracking as a result of crossing fibers (bilateral cingulum hippocampus and arcuate fasciculi). AFQ is composed of three major steps: 1) estimation of whole-brain fiber tractography; 2) segmentation of fiber tracts; and 3) refinement of fiber tracts. In the first step, whole-brain fiber tractography was estimated using a deterministic streamlines tracing algorithm (STT) [5, 6] with a fourth-order Runge-Kutta path integration method with 1-mm step size. Voxels with FA greater than 0.3 were seeded. Streamlines were traced bidirectionally along the principal diffusion axis, with stopping criteria including FA less than 0.2 or an angle between steps greater than 30°. The resulting whole-brain tractography was used for segmentation of fiber tracts, using a waypoint ROI-based method [7]. Fibers were identified that passed through pairs of waypoint ROIs. These waypoint ROIs are placed in order to identify fascicles that pass through stereotypical locations. AFQ uses waypoint ROIs drawn on an MNI template that are subsequently non-linearly warped to each participant’s brain [1]. Refinement of segmented fiber tracts is achieved by comparing candidate fibers to a fiber tract probability map created by Hua et al. (2008) [8]. This fiber tract probability map indicates the probability that a voxel is associated with a given fiber tract. Estimated fibers are discarded if they exhibit aberrant trajectories associated with fiber tract low probabilities. Fiber tracts are then cleaned by iteratively discarding individual outlier fibers as defined by their distance from fiber tract core or length compared to the average fiber. FA was then assessed along each fiber group core using spline interpolation. A single fiber’s contribution to the core estimate is weighted based on the likelihood that the fiber is a member of the given fiber tract, computed as the Mahalanobis distance. The mean FA was then computed for each fiber tract on an individual subject basis.

*MDP: MRI Preprocessing and Tractography Estimation*

Eddy and motion correction were completed using FSL’s *eddy* tool. Whole-brain tractography was estimated using an optimized global probabilistic tractography method [9]. FA was estimated from the single-tensor diffusion model [3] and on a voxel-wise basis this information is used a prior probability for seed point locations (i.e., seed points were chosen randomly throughout the white matter based on their proportional FA). At each voxel, the global tractography method uses directionality information that is parameterized by the given voxel’s orientation distribution function (ODF). ODFs were estimated using Q-ball imaging [10]. For an identified seed point, the algorithm selects fibers by generating scores for approximately 1,000,000 candidate curves. The curve that is scored highest is retained in the final fiber set as described by the following:

(1)

Where *s* is arc length (with values ranging from to ), is the ODF value at location and direction (i.e., the unit tangent). For a given subject, 35,000 fibers were estimated, thus comprising the whole-brain fiber tractography for that subject. We used the Automatic Registration Toolkit (ART) [11, 12] to nonlinearly transform the high-resolution T1-weighted images to native subject diffusion space.

*MDP: Identification and Registration of Maximum Density Paths*

We here describe the MDP procedure (for further detail see Prasad et al. ). Fifty white matter ROIs from the Johns Hopkins University white matter atlas [13] were warped to each individual’s DWI space. All fibers intersecting an ROI were identified, resulting in fiber clusters. Each fiber of a fiber cluster was then scored based on the number of ROI voxels it intersects and fibers with low intersection scores were discarded. For a given ROI, a density image is created based on the number of fibers that intersect a given voxel. After smoothing, from this density image a graph is created with voxels as nodes, with each node having 26 undirected edges specified as a weight calculated from the sum of voxel densities:

(2)

Where represents the density at location (i.e., at node *k*). Then, in order to represent high density edges as low values to facilitate the identification of optimal paths, edge weights were subtracted the maximum initial edge cost. Next, Dijkstra’s algorithm [14] was used to find the highest density path between seed point pairs. A total of 67 seed pairs were obtained from a set of previously identified points in the white matter ROI atlas. The resulting paths were subsequently smoothed using a Gaussian kernel in order to facilitate registration. This process resulted in a total of 67 compact representations of important white matter paths, namely, MDPs.

In order to compare the 67 MDPs from the 50 ROIs across subjects, MDPs were registered using a geodesic curve registration procedure [15, 16]. This procedure computes a mean MDP across subjects using shape matching enabled by geodesics. The geodesic represents the shortest length of paths in an infinite-dimensional space of curves, induced by Riemannian metrics based on the tangent space. The geodesic also indexes the optimal geometric differences between curves that can be used for co-registration. The mean MDP was used as a registration target for the elastic shape matching of subject MDPs, thus allowing for point-wise statistical comparisons of FA along each MDP.

**Supplemental Information References**

1. Friston KJ, Ashburner J: **Generative and recognition models for neuroanatomy**. *Neuroimage* 2004, **23**:21–24.

2. Chang L-C, Jones DK, Pierpaoli C: **RESTORE: Robust estimation of tensors by outlier rejection**. *Magn Reson Med* 2005, **53**:1088–1095.

3. Basser PJ, Pierpaoli C: **Microstructural and Physiological Features of Tissues Elucidated by Quantitative-Diffusion-Tensor MRI**. *J Magn Reson B* 1996, **111**:209–219.

4. Yeatman JD, Dougherty RF, Myall NJ, Wandell BA, Feldman HM: **Tract Profiles of White Matter Properties: Automating Fiber-Tract Quantification**. *PLoS ONE* 2012, **7**:e49790.

5. Mori S, Crain BJ, Chacko VP, Van Zijl P: **Three‐dimensional tracking of axonal projections in the brain by magnetic resonance imaging**. *Ann Neurol* 1999, **45**:265–269.

6. Basser PJ, Pajevic S, Pierpaoli C, Duda J, Aldroubi A: **In vivo fiber tractography using DT‐MRI data**. *Magn Reson Med* 2000, **44**:625–632.

7. Wakana S, Caprihan A, Panzenboeck MM, Fallon JH, Perry M, Gollub RL, Hua K, Zhang J, Jiang H, Dubey P, Blitz A, Van Zijl P, Mori S: **Reproducibility of quantitative tractography methods applied to cerebral white matter**. *Neuroimage* 2007, **36**:630–644.

8. Hua K, Zhang J, Wakana S, Jiang H, Li X, Reich DS, Calabresi PA, Pekar JJ, van Zijl PCM, Mori S: **Tract probability maps in stereotaxic spaces: Analyses of white matter anatomy and tract-specific quantification**. *Neuroimage* 2008, **39**:336–347.

9. Prasad G, Nir TM, Toga AW, Thompson PM: **Tractography Density and Network Measures in Alzheimer's Disease**. *Proc IEEE Int Symp Biomed Imaging* 2013:692–695.

10. Aganj I, Lenglet C, Sapiro G, Yacoub E, Ugurbil K, Harel N: **Reconstruction of the Orientation Distribution Function in Single- and Multiple-Shell q-Ball Imaging Within Constant Solid Angle**. *Magn Reson Med* 2010:554–566.

11. Klein A, Andersson J, Ardekani BA, Ashburner J, Avants B, Chiang M-C, Christensen GE, Collins DL, Gee J, Hellier P, Song JH, Jenkinson M, Lepage C, Rueckert D, Thompson P, Vercauteren T, Woods RP, Mann JJ, Parsey RV: **Evaluation of 14 nonlinear deformation algorithms applied to human brain MRI registration**. *Neuroimage* 2009, **46**:786–802.

12. Ardekani BA, Guckemus S, Bachman A, Hoptman MJ, Wojtaszek M, Nierenberg J: **Quantitative comparison of algorithms for inter-subject registration of 3D volumetric brain MRI scans**. *J Neurosci Methods* 2005, **142**:67–76.

13. Wakana S, Jiang H, Nagae-Poetscher LM, van Zijl PCM, Mori S: **Fiber Tract-based Atlas of Human White Matter Anatomy**. *Radiology* 2004, **230**:77–87.

14. Dijkstra EW: **A note on two problems in connexion with graphs**. *Numer Math* 1959, **1**:269–271.

15. Joshi SH, Klassen E, Srivastava A, Jermyn I: **A Novel Representation for Riemannian Analysis of Elastic Curves in Rn**. In *Proc IEEE Comput Soc Conf Comput Vis Pattern Recognit*. IEEE; 2007:1–7.

16. Joshi SH, Klassen E, Srivastava A, Jermyn I: **Removing Shape-Preserving Transformations in Square-Root Elastic (SRE) Framework for Shape Analysis of Curves**. *Proc IEEE Comput Soc Conf Comput Vis Pattern Recognit* 2007, **4679**:387–398.
